# Supplementary material for: Computational ranking identifies Plexin-B2 in circulating tumor cell clustering with monocytes in breast cancer metastasis
Source: Nat Commun. 2025 Aug 16;16:7649. doi: 10.1038/s41467-025-62862-z (PMC12357858; doi:10.1038/s41467-025-62862-z)
Supplement: Supplementary file 1 — Supplementary Information [file 41467_2025_62862_MOESM1_ESM.pdf]

Computational ranking identifies Plexin-B2 in circulating tumor cell clustering with monocytes in breast cancer metastasis

Emma Schuster[1,2#], Nurmaa K. Dashzeveg[1#], Fangjia Tong[1#], Yuzhi Jia[1], Lamiaa El-Shennawy[1], Tong Zhang[3], Andrew D. Hoffman[1,4], Reta Birhanu Kitata[3], Golam Kibria[1],Youbin Zhang[5], Joshua R. Squires[1], Chunlei Zheng[6,], Erika Ramos[1], Rokana Taftaf[1], David Scholten[1,2], Hannah F. Almubarak[1,2], Valery Adorno-Cruz[1], David P. Sullivan[7], Carolina Reduzzi [8], Allegra C. Minor[2,9], William Purev-Ochir[1], Sabina Spahija[5], Rong Xu[6], Kalliopi P. Siziopikou[7,9], Leonidas C. Platanias[5,9], Ami Shah[5,9], William A. Muller[7,9], William J. Gradishar[5,9], Massimo Cristofanilli[8], Chia-Feng Tsai[3*], Tujin Shi[3*], & Huiping Liu[1,5,9,10,11*]

[1]Department of Pharmacology, Northwestern University Feinberg School of Medicine, Chicago, IL, USA
[2]Driskill Graduate Program in the Life Sciences, Northwestern University Feinberg School of Medicine, Chicago, IL, USA
[3]Biological Sciences Division, Pacific Northwest National Laboratory, Richland, WA, USA
[4]ExoMira Medicine Inc, Chicago, IL, USA
[5]Division of Hematology and Oncology, Department of Medicine, Northwestern University Feinberg School of Medicine, Chicago, IL, USA
[6]Center for Artificial Intelligence in Drug Discovery, Case Western Reserve University, Cleveland, OH, USA
[7]Department of Pathology, Northwestern University Feinberg School of Medicine, Chicago, IL, USA
[8]Division of Hematology and Medical Oncology, Department of Medicine, Weill Cornell School of Medicine, New York, NY, USA
[9]Department of Biochemistry and Molecular Genetics, Northwestern University Feinberg School of Medicine, Chicago, IL, USA
[10]Robert H. Lurie Comprehensive Cancer Center, Northwestern University Feinberg School of Medicine, Chicago, IL, USA
[11]Chan Zuckerberg Biohub Chicago, Chicago, IL, USA.

Corresponding authors: Huiping Liu, MD, PhD, Northwestern University, Email: huiping.liu@northwestern.edu (Lead Contact); Tujin Shi, PhD, Pacific Northwest National Laboratories, Email: Tujin.Shi@pnnl.gov.
#These authors contributed equally.
*These authors jointly supervised this work.

Supplementary Information

1. Supplementary Tables S1
2. Supplementary Figure S1-S9

Supplementary Table S1. Biological processes for proteins that were significantly up-regulated in *PB2* KD cancer cells.

GO Term (Biological Process)	Fold Change
astrocyte activation involved in immune response (GO:0002265)	37.85
positive regulation of amyloid fibril formation (GO:1905908)	28.39
glutamate biosynthetic process (GO:0006537)	22.71
positive regulation of artery morphogenesis (GO:1905653)	22.71
regulation of artery morphogenesis (GO:1905651)	22.71
sequestering of calcium ion (GO:0051208)	22.71
negative regulation of metallopeptidase activity (GO:1905049)	21.63
positive regulation of protein catabolic process in the vacuole (GO:1904352)	18.92
common-partner SMAD protein phosphorylation (GO:0007182)	16.22
wound healing involved in inflammatory response (GO:0002246)	16.22
positive regulation of pinocytosis (GO:0048549)	16.22
positive regulation of receptor clustering (GO:1903911)	16.22
positive regulation by symbiont of entry into host (GO:0075294)	15.14
positive regulation of viral entry into host cell (GO:0046598)	15.14
negative regulation of fibrinolysis (GO:0051918)	14.56
regulation of basement membrane organization (GO:0110011)	13.76
fatty acid transmembrane transport (GO:1902001)	12.62
regulation of protein catabolic process in the vacuole (GO:1904350)	12.62
astrocyte activation (GO:0048143)	11.83
positive regulation of integrin-mediated signaling pathway (GO:2001046)	11.65
carnitine metabolic process (GO:0009437)	11.65
low-density lipoprotein particle clearance (GO:0034383)	10.81
positive regulation of viral life cycle (GO:1903902)	10.6
regulation of fibrinolysis (GO:0051917)	10.51
endoplasmic reticulum tubular network organization (GO:0071786)	10.09
relaxation of cardiac muscle (GO:0055119)	10.09
regulation of metallopeptidase activity (GO:1905048)	10.09
regulation of receptor clustering (GO:1903909)	10.09
positive regulation of superoxide anion generation (GO:0032930)	9.46
regulation of steroid hormone biosynthetic process (GO:0090030)	9.46
positive regulation of T cell receptor signaling pathway (GO:0050862)	9.46
amyloid precursor protein catabolic process (GO:0042987)	9.46
positive regulation of immune response to tumor cell (GO:0002839)	9.46
positive regulation of response to tumor cell (GO:0002836)	9.46
plasma lipoprotein particle clearance (GO:0034381)	9.46
regulation of membrane protein ectodomain proteolysis (GO:0051043)	9.08
regulation of integrin-mediated signaling pathway (GO:2001044)	9.01
myeloid dendritic cell differentiation (GO:0043011)	9.01
regulation of macrophage cytokine production (GO:0010935)	8.96

Supplementary Figure S1

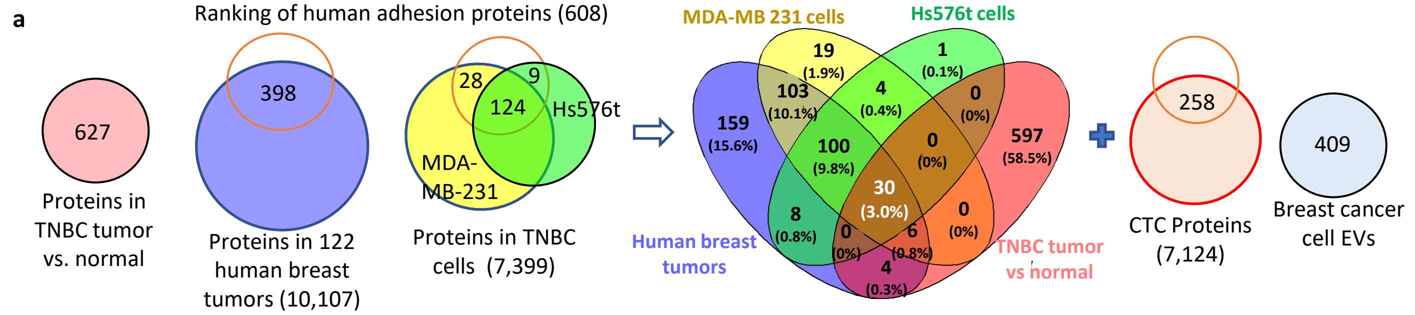

b

Final rank	Protein
1	PLXNB2
2	PPIA
3	EZR
4	ACTB
5	HMGB1
6	CLIC1
7	F11R
8	HSPH1
9	TLN1
10	TJP2
11	SRC
12	LGALS1
13	PDLIM1
14	PRKAR1A
15	TMOD3
16	RHOA
17	DUSP3
18	HBB
19	BSG
20	RIC8A
21	ANXA1
22	DSC2
23	FERMT3
24	RAC2
25	PPP1CA
26	CD44
27	MSN
28	SERPINF2
29	PYCARD
30	RAP2B

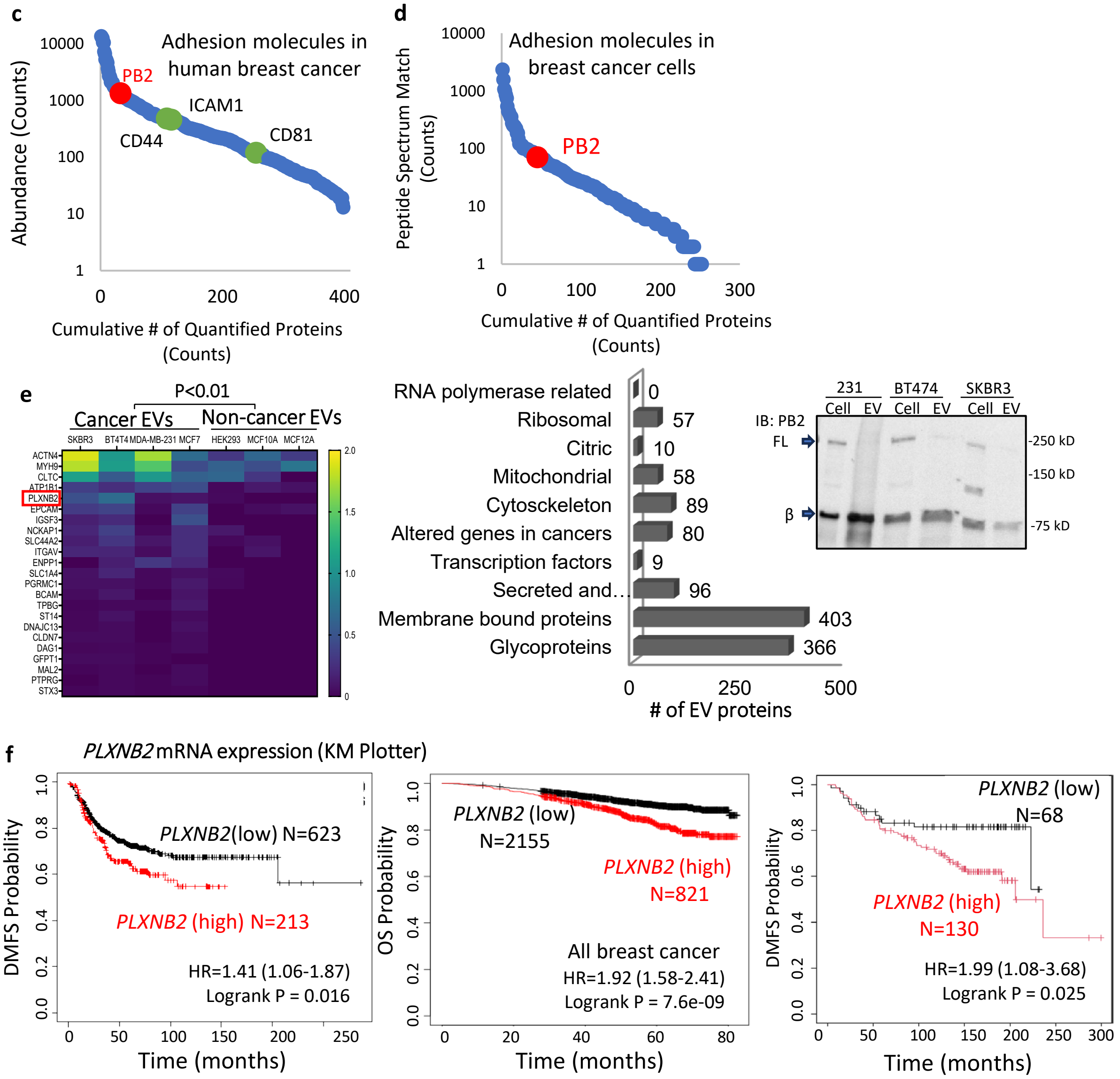

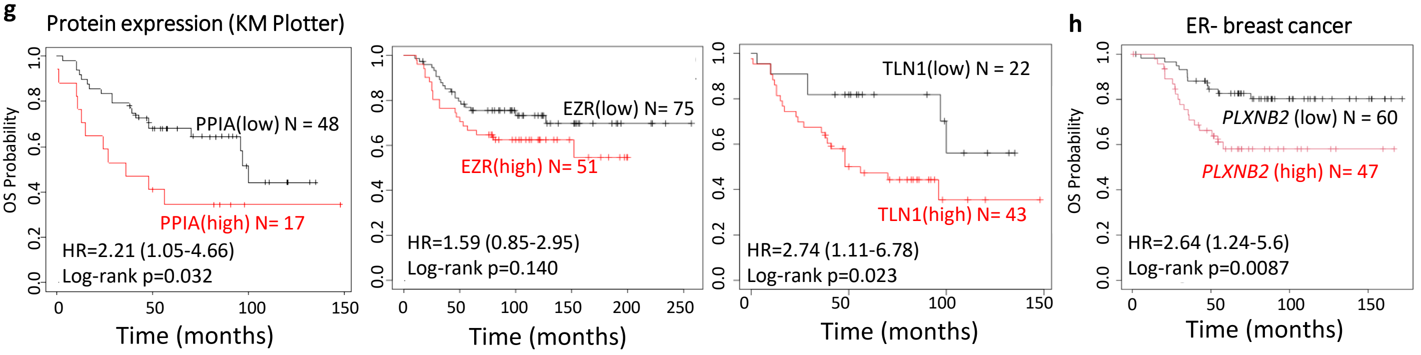

Supplementary Figure S1. Mathematical modeling analysis of adhesion protein ranking

a) The workflow of integrating computational rankings of adhesion proteins into a four-way Venn diagram to identify novel candidates associated with patient outcomes based on protein abundance measured by MS-based global proteomics in primary breast tumors (N=122) and TNBC cell lines, altered protein expression in TNBC tumor vs. normal tissue voxels, to identify 30 overlapping candidates for patient outcome association analysis. Those were further analyzed in the mass spectrometry proteomic data of human CTCs and breast cancer cell-specific EVs.

b) Expanded table of top hits from combined database analysis using the mathematical model for R-score shown in Fig 1A for ranking to identify top adhesion proteins in TNBC with overall R score and rank; lower overall R-scores imply higher significance across multiple datasets

c) Average spectral counts-based abundance ranking of 398 identified adhesion/surface proteins across 122 treatment-naïve primary breast patient samples (N=122) (https://doi.org/10.1016/j.cell.2020.10.036)[32], highlighting key proteins previously identified to be significant in CTCs (CD44, ICAM1, CD81 in green) and a novel candidate PLXNB2 (PB2) at a higher rank (red).

d) Average peptide spectrum match (PSM)-based abundance ranking of 252 identified adhesion/surface proteins in MDA-MB-231 triple-negative breast cancer (TNBC) cell lines (N=3) (https://doi.org/10.1021/acs.jproteome.1c00293)[33].

e) Left panel: Heat map of MS-identified differentially expressed proteins between EVs derived from breast cancer cell lines vs. EVs from immortalized normal epithelial cells (unpaired t-test $P<0.01$). PLXNB2 is indicated by a red box. Middle panel: Categories of MS-identified EV proteins (total 1603 peptides) from normal and cancer cell-secreted EVs. Right panel: immunoblot of PLXNB2 (PB2) using cellular lysates and EV specimens derived from MDA-MB-231, BT474, and SKBR3 cells. The full-length (FL) and truncated PLXNB2 proteins are arrow appointed to be around 200 KD and 75 kD, respectively.

f) KM plots for distant metastasis-free survival (DMFS) of grade-3 breast cancer (N=836) with high vs. low *PLXNB2* mRNA expression (data from KMPlotter), OS of all breast cancer (N=2976) with high vs. low *PLXNB2* mRNA expression (data from KMPlotter), DMFS of all breast cancer (N=198) with high vs. low *PLXNB2* mRNA expression using data from GEO (GSE7390), separated by best cut-off values. P-values were calculated using log rank test.

g) Representative KM plots of OS probability in breast cancer based on protein expression of PPIA, EZR, and TLN1 among the top 30 adhesion molecule candidates, the groups with high and low protein expressions were determined by best cut-off via KMplotter.

h) KM plots for OS of ER- breast cancer with high vs. low *PLXNB2* mRNA expression using data from GEO (GSE58812), N=107, separated by best cut-off values. P-values were calculated using a log-rank test. Source data are provided as a Source Data file.

Supplementary Figure S2

a

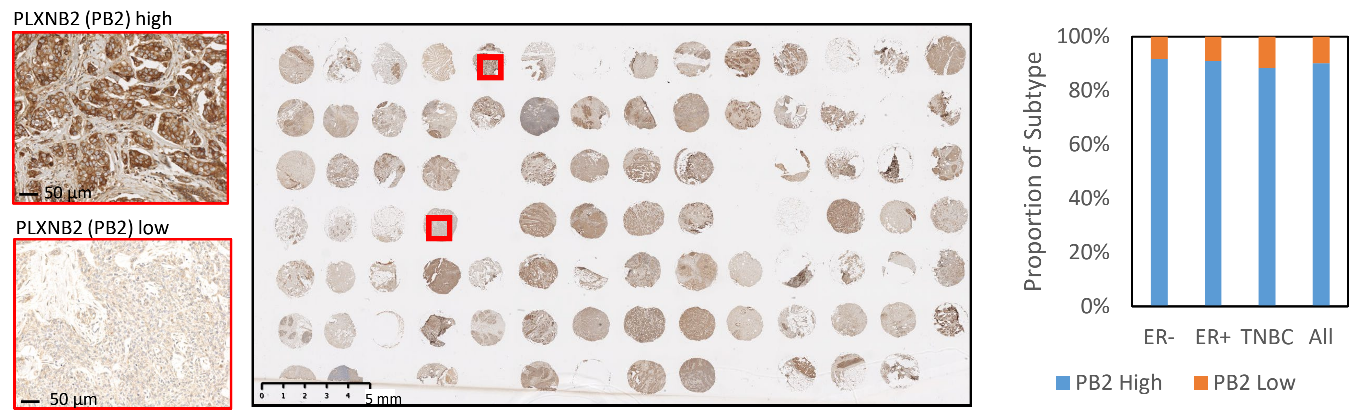

b

	PLXNB2 High	PLXNB2 Low
ER-	11	1
ER+	20	2
TNBC	23	3
All	55	6

*P=0.042

Met	PLXNB2 High	PLXNB2 Low
Brain	2/11	0/4
Lung	5/11	2/4
Bone	7/11	1/4
Liver	6/11	0/4

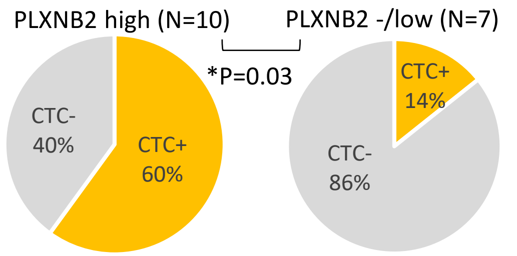

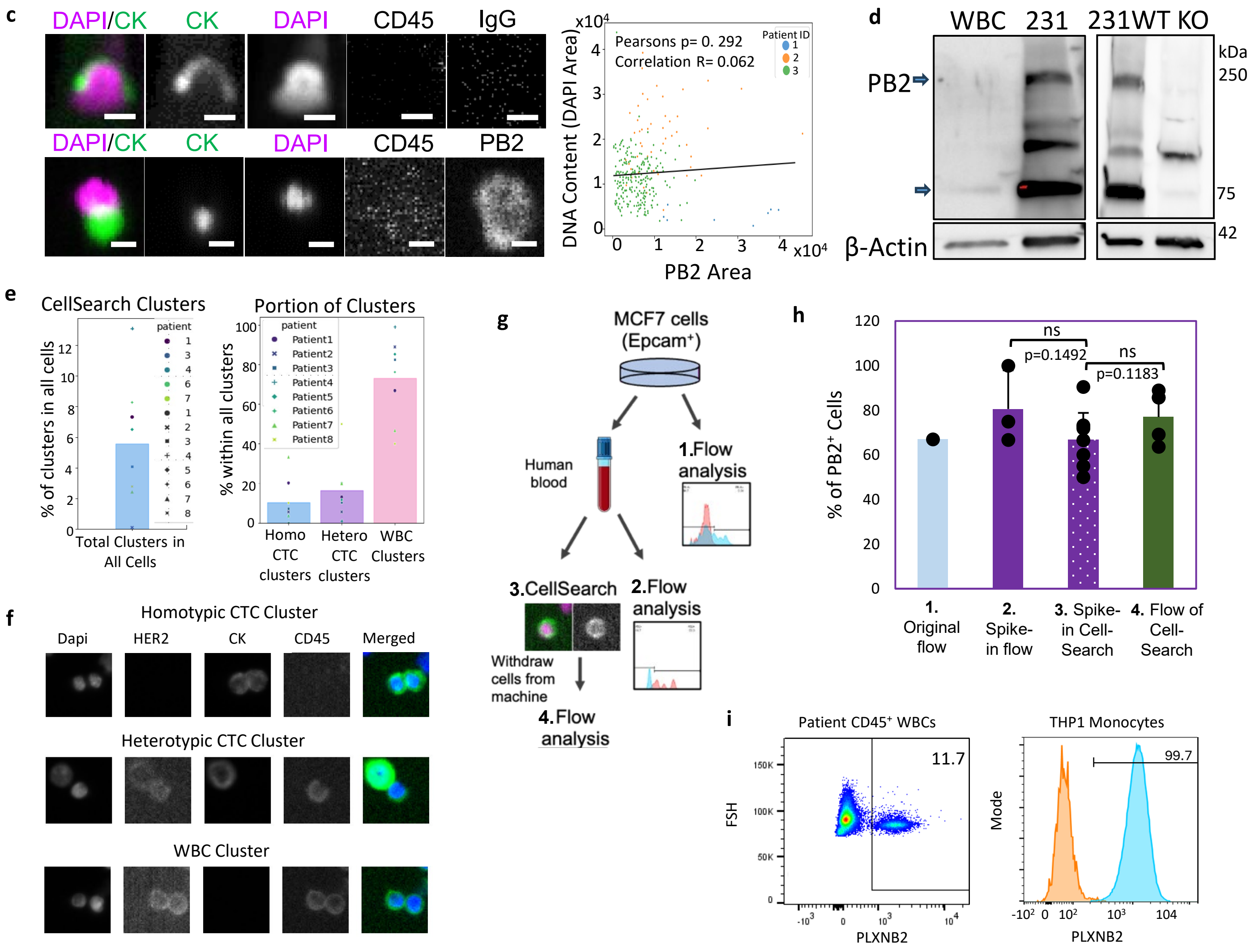

Supplementary Figure S2. PLXNB2 expression in human breast tumors is associated with detection of CTCs.

a) Left panels: PLXNB2 IHC images of representative PLXINB2 high and low expression and the patient tissue microarray of advanced breast cancer specimens (N=86). Samples with > 10% positive tumor regions were called positive or PLXNB2-high. The rest samples were PLXNB2-low including those between 1-10% positive (weak positive) and those with <1% positive as negative. The overall low intensity shown in PLXNB2 staining was also assigned as low. Right panel: Proportion (%) of PLXNB2 (PB2) high and low tumors in the TMA stratified by the receptor expression status of breast cancer via IHC staining.

b) Left tables: Summary of PLXNB2 status in the TMA cases, stratified by receptor expression and metastatic organs in breast cancer. Right pie panels: CTC detection within the patients from the Northwestern B06 cohort with PLXNB2 high or negative/low tumors.

c) Left panels: Cell Search images of IgG isotype negative control staining (0/18 CTCs positive) and anti-PB2 staining (13/25 CTCs positive) with the blood cells from the identical breast cancer patient sample. Scale bar = 5 μm. **Right panel:** dot plot of CTCs with signals of DAPI-based DNA content and PB2 expression (patient N=3, Pearson p=0.292, and R=0.062).

d) Immunoblot images for PLXNB2 (PB2) in the lysates of patient white blood cells, and MDA-MB-231 cells (WT and *PB2*-KO).

e-f) Quantified CellSearch-detected clusters (total, homotypic CTC clusters, heterotypic CTC clusters, and WBC clusters) (e) and images (f) within fixed blood cells of cancer patients (N=8).

g-h) Experimental workflow (g): MCF7 cells (2x104) were added into freshly collected human blood (15 ml) which was then divided into two tubes (2x7.5 ml). One blood tube was loaded directly to CellSearch for CTC and PLXNB2 (PB2) analysis and another was processed for flow analysis of white blood cells (WBCs). The CTCs imaged via CellSearch were retrieved for flow validation. Quantification (h) of PB2+ tumor cells analyzed by flow cytometry (1. pre-spike in, 2, post-spike into the blood, 4. retrieved cells from CellSearch) and CellSearch imaging (3).

i) Flow analyses of PLXNB2 in human PBMCs and THP1 monocytes.

P values were calculated using one-sided ANOVA. Source data are provided as a Source Data file.

Supplementary Figure S3

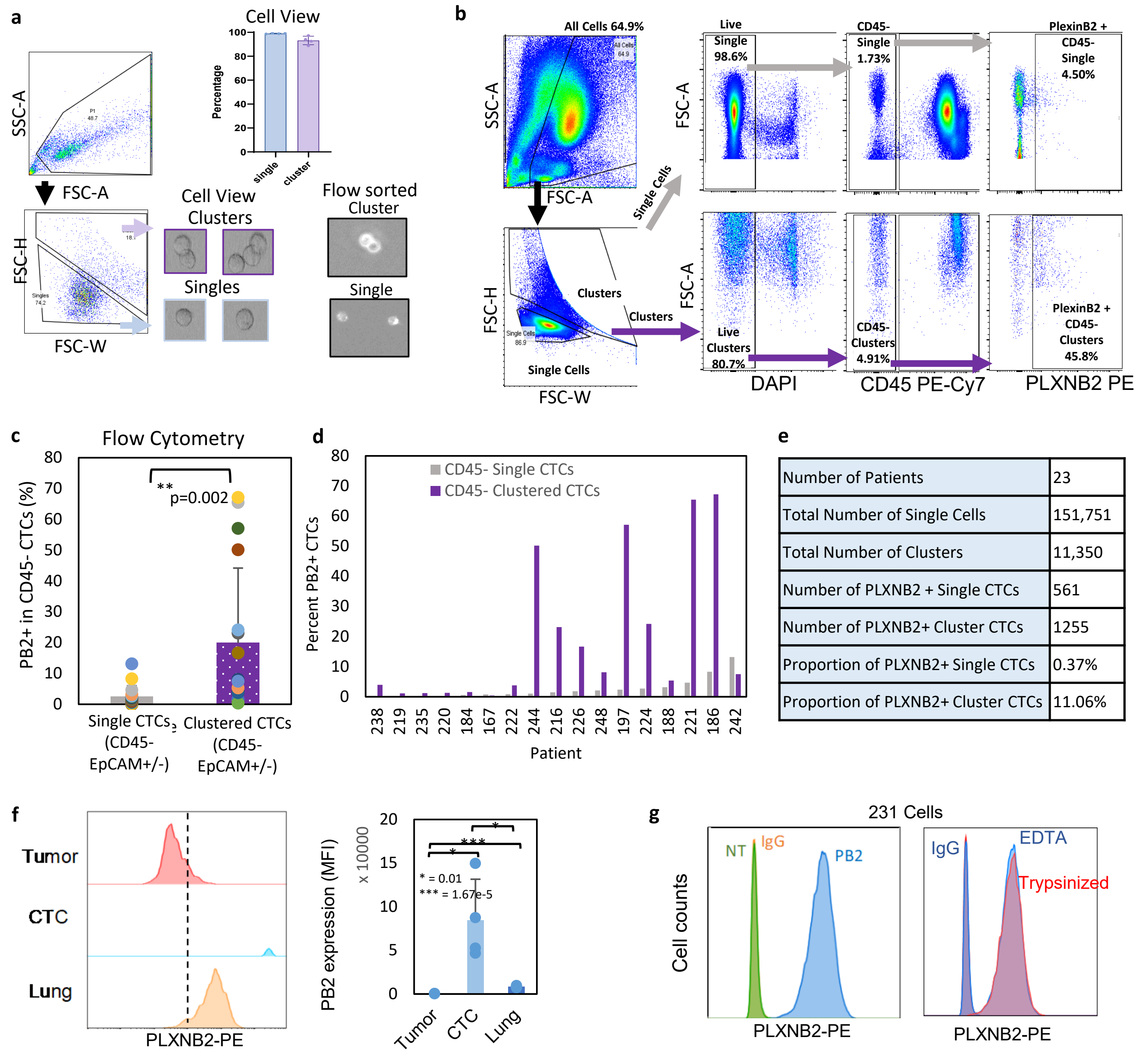

Number of Patients	23
Total Number of Single Cells	151,751
Total Number of Clusters	11,350
Number of PLXNB2 + Single CTCs	561
Number of PLXNB2+ Cluster CTCs	1255
Proportion of PLXNB2+ Single CTCs	0.37%
Proportion of PLXNB2+ Cluster CTCs	11.06%

Supplementary Figure S3. PLXNB2 expression analysis in patient CTCs via flow cytometry.

a) Validations of gated singles and clusters on flow channels SSC-A/FSC-A and FSC-H/FSC-W via two methods (1) BD CellView imaging with representative images (top middle panels) and assessment of 1,000 events (99% singles and 93% clusters, N=3) (top right panel), and (2) Representative microscopy images of sorted singles and clusters via BD Aria.

b) Representative flow cytometry gating of single and clustered blood cells, by $PB2^{+/-}$ and $CD45^{+/-}$ status, from the B06 cohort of advanced breast cancer patients.

c) Average percentage of cells expressing PLXNB2 (PB2) in $CD45^{-}$ single and clustered CTCs from flow analysis, including all $EpCAM^{+/-}$ CTCs, N=17 patients, data reported as mean values +/- SD, p-value from a two-sided unpaired t-test.

d) Percentage of $PB2^{+}$ $CD45^{-}$ single and $CD45^{-}$ clustered CTCs from advanced breast cancer patients at the time of blood draw, N=17 patients, data stratified by individual patient.

e) Table showing the total number and proportion of CTC single cells or CTC clusters that are $PB2^{+}$ from breast cancer patients as reported by flow analysis, N=23.

f) Flow cytometry analysis of PB2 expression (mean fluorescence intensity, MFI) in dissociated primary tumor cells (tumor), CTCs, and lung metastasis (lung) from L2G-labeled TNBC PDX mice. N=4 mice. P-values were calculated using one-sided ANOVA.

g) Flow cytometry analysis of distinct PB2 expression in contrast to IgG isotype control (left panel) and similar profiles between EDTA-dissociated and trypsinized MDA-MB-231 cells. Source data are provided as a Source Data file.

Supplementary Figure S4

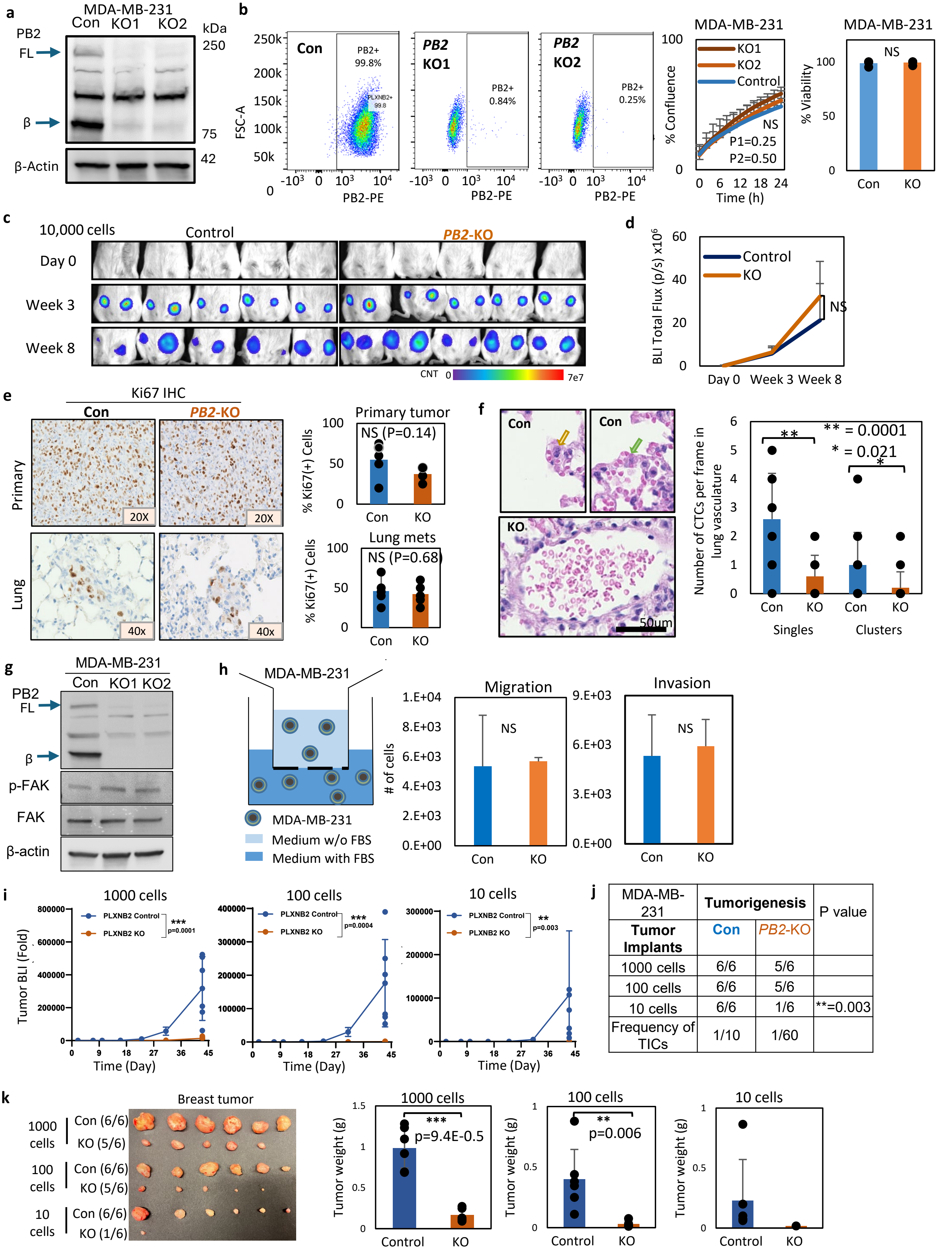

j

MDA-MB-231	Tumorigenesis		P value
Tumor Implants	Con	PB2-KO	
1000 cells	6/6	5/6	
100 cells	6/6	5/6	
10 cells	6/6	1/6	**=0.003
Frequency of TICs	1/10	1/60	

Supplementary Figure S4. *PB2* KO inhibits CTC cluster formation in spontaneous metastasis of TNBC.

a) Immunoblots of full-length (FL) and truncated (Tr) PLXNB2 (PB2) in MDA-MB-231 cells with two pooled clones of CRISPR/Cas9-mediated KO, KO1 and KO2, with PB2-specific bands at 250 kDa (full length) and 75 kDa (cleaved β subunit), N=3 experiments.

b) Left panels: flow cytometry dot plots of MDA-MB-231 Cas9 control cells for PB2, and pooled knockout clones KO1, and KO2. Right panels: proliferative confluence via IncuCyte imaging and viability of the WT and KO cells via DAPI staining and flow cytometry (N=3). An unpaired two-sided t-test was used to evaluate differences between WT and KO groups. NS=not significant.

c) Bioluminescence images of tumor growth of 10,000 MDA-MB-231 cells of Control and *PB2* KO1, labeled with L2G luciferase reporter in NSG mice over 8 weeks, two injections at L4/R4 mammary fat pads per mice, N=5 mice/group.

d) Average ROI counts of bioluminescence imaging from 10,000 cell-tumor implants as measured by an SII Lago imager, data reported as mean +/-SD over time, N=5 mice, p-value from an unpaired two-sided t-test. NS = not significant.

e) Ki67 IHC images (left panels) and quantification (right panels) of MDA-MB-231 tumors (Control and *PB2*-KO) and mouse lungs bearing the spontaneous metastases of these tumors, estimated as the percentage of cells with Ki-67-positive nuclear immunostaining in the WT/KO primary tumor cells (NS, p=0.12) and lung metastasis (NS, p=0.65) after 10 weeks of implantations, N=5 mice. NS= not significant.

f) Left panels: images of the lungs (stained with H&E) collected from the mice bearing *PB2* WT and KO tumors with spontaneous metastases. Right panel: CTCs were quantified in the vascular structures shown in the lung images; data reported as mean +/- SD over time, N=5 mice (data points = 6, 3, 4, and 3 image fields for the four groups, respectively). P-values were calculated using ANOVA.

g) Immunoblots showing expression of FAK and p-FAK in control and *PB2* KO MDA-MB-231 cells.

h) Transwell migration and invasion analyses of control and *PB2* KO MDA-MB-231 cells. Cells migrated into FBS-containing media or invaded growth factor-reduced Matrigel after 24 hours were measured by IncuCyte imaging, N=3 experiments, data reported as mean +/- SD, a two-sided unpaired t-test p-value >0.05 (NS).

i-k) Tumorigenesis measured via bioluminescence imaging (BLI) (i), palpable tumors (j) and tumor weight (k) with serial dilutions of L2T-labeled MDA-MB-231 cells of Control and *PB2* KO1 (1,000 cells, 100 cells, and 10 cells) for orthotopic implantations (N=6) at NSG mouse mammary fat pads. BLI signals over 6 weeks were compared via t-test (i). The palpable tumors and tumor weights of two groups were compared via an unpaired two-sided t-test (j and k). Source data are provided as a Source Data file.

Supplementary Figure S5

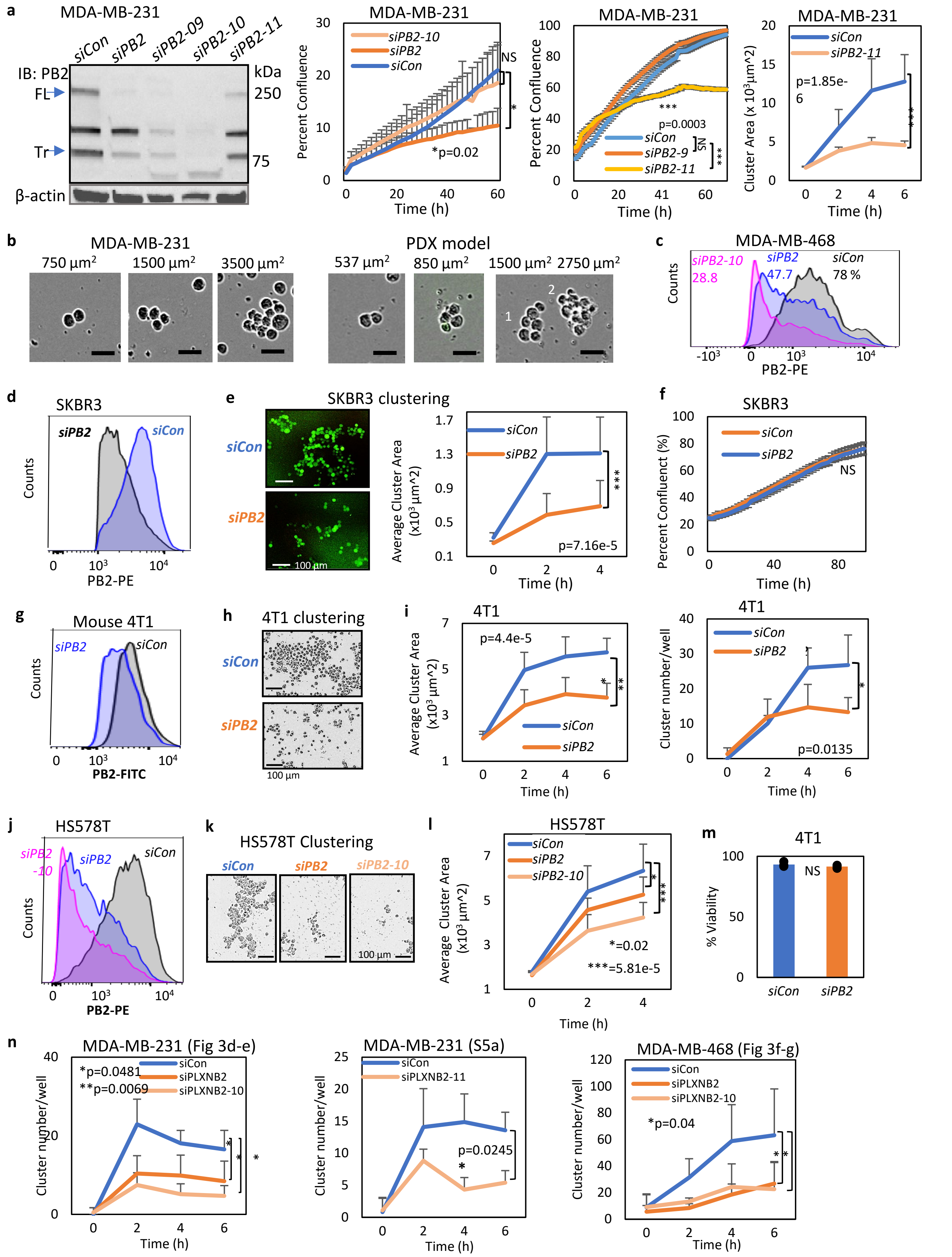

Supplementary Figure S5. ***PLXNB2*** **knockdown inhibits clustering of human and mouse breast cancer cells.**

a) Left panel: Western blot showing MDA-MB-231 KD efficiency of *PLXNB2* (*PB2*) using SmartPool (*siPB2*) siRNA as well as single siRNAs (si09, si10, si11), N=3 experiments; **Middle Panels:** Cell growth (confluence) assay showing *siPB2* SmartPool and *siPB2-10, 9, 11* single siRNA KD with at least 3 technical replicates, NS=0.13, N=3 experiments; **Right Panel:** Cluster size curves of MDA-MB-231 control and *siPB2-11* knockdown cells with at least 3 technical replicates.

b) Representative images of clusters of PDX (left) and cells lines-MDA-MB-231 (right) cells taken by Incucyte imager. Scale bar = 30 µm

c) Flow analysis showing MDA-MB-468 KD of *PB2* using SmartPool (*siPB2*) siRNA as well as single siRNA (*siPB2-10*), N=3 experiments.

d) Flow panel showing KD efficiency of SKBR3 breast cancer cells after double transfection with *PB2* SmartPool siRNA.

e) Representative images (**left**) of SKBR3 cell clusters after 4 h as taken by the IncuCyte Live Cell Imager and quantification (**right**) of cluster area of SKBR3 cells with *PB2* KD after 4 h, average cluster area measured by IncuCyte, with N=4 experiments

f) Proliferation-related confluence of SKBR3 cells showing *siPB2* SmartPool KD with at least 3 technical replicates.

g) Flow panel showing KD efficiency of 4T1 mouse breast cancer cells after double transfection with *PB2* SmartPool siRNA.

h) Representative images of 4T1 cell clusters after 6 h as taken by the Incucyte Live Cell Imager.

i) Quantification of clustering assay of 4T1 cells with *PB2* KD after 6 h, average cluster area measured by Incucyte, with N=3 experiments.

j) Flow panel showing KD efficiency of HS578T breast cancer cells after double transfection with *PB2* SmartPool siRNA and *siPB2-10* single *PB2* siRNA.

k) Representative images of HS578T cell clusters after 4 h as taken by the Incucyte Live Cell Imager.

l) Quantification of clustering assay of HS578T cells (+/- *siPB2* KD) after 4 h, average cluster area measured by Incucyte, with N=3 experiments.

m) Viability as measured by DAPI staining and flow cytometry analysis of 4T1 cells after double transfection with *PB2* SmartPool siRNA, N=3.

n) Quantified numbers of homotypic tumor clusters (top row) in MDA-MB-231 (Fig 3d-e, S5a) and MDA-MB-468 cells (Fig 3f-g) (+/-*PB2* KD, smart pool or individual siRNA-10, -11) over 6 h, measured by Incucyte imaging, with N=3-5 experiments.

Data are presented as mean values ± SD. P-values between two groups were calculated using two-sided unpaired t-tests. For comparisons involving more than two groups, one-sided ANOVA was used. Source data are provided as a Source Data file.

Supplementary Figure S6

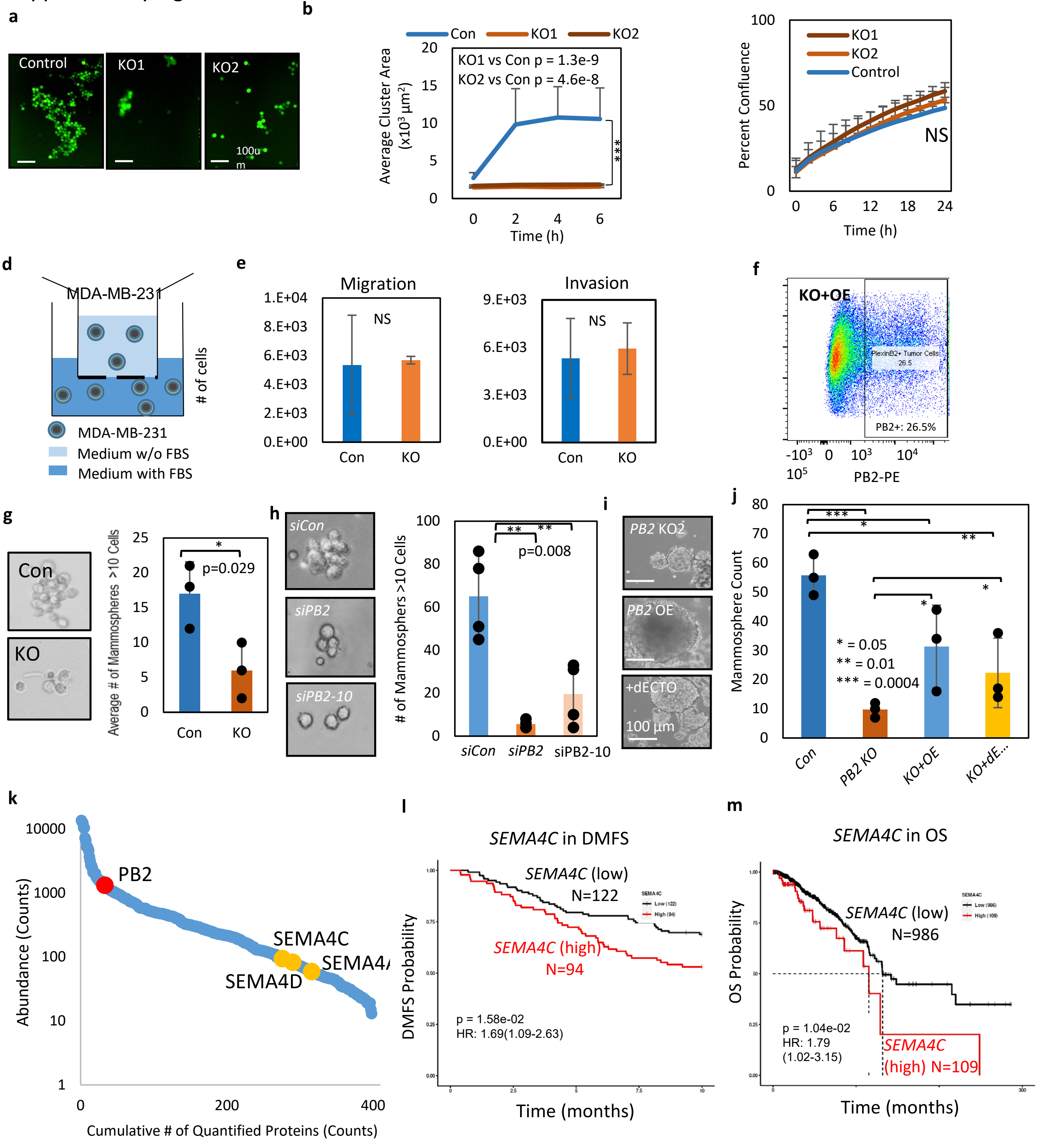

Supplementary Figure S6. PLXNB2 KO inhibits tumor cell clustering and mammosphere formation.

a-b) Representative images (a) and cluster size quantification (b) of MDA-MB-231 *PB2* control, KO1, and KO2 cell clusters after 6 h as taken by the IncuCyte Live Cell Imager; N=3 experiments with at least 3 technical replicates each.

c) Proliferation assay of MDA-MB-231 control and KO cells over 24 h measured as percent confluence by IncuCyte, N=3 experiments, data reported as mean +/- SD, p-values were calculated using ANOVA, p(Con v KO1) = 0.25, p(Con v KO2) = 0.5.

d-e) Transwell migration and invasion analyses of MDA-MB-231 *PB2* WT and KO tumor cells. Cells migrated to FBS-containing media after 24 hours were measured by IncuCyte, N=3 experiments, data reported as mean +/- SD, a two-sided unpaired t-test p-value >0.05 (NS).

f) Flow cytometry validation of overexpressed Plexin B2.

g) Mammosphere formation images (left) and counts (right panels) of MDA-MB-231 *PB2* control and KO cells after 8 days using Mammary Stem Cell medium, N=3. Mammospheres larger than 10 cells were counted for quantification.

h) Representative images (left) of average mammosphere counts of 10 cells or more (right) of MDA-MB-231 WT cells with scramble (*siCon*), *PB2* SmartPool (*siPB2*), and *PB2* single (*siPB2-10*) siRNA KD after 7 days using MammoCult™ Human Medium Kit (STEMCELL Technologies, Catalog# 05620); N=3 experiments with at least 5 technical replicates each. P-values were calculated using ANOVA.

i-j) Mammosphere formation images (i) and quantification (j) of MDA-MB-231 WT control (Con) cells, *PB2* KO cells, and KO cells with *PB2* rescue by overexpression with full length (OE) and the mutant depleting the extracellular domain (dECTO) for 14 days using MammoCult™ Human Medium Kit, N=4 replicates. Mammospheres larger than 100 microns across were counted. P-values were calculated using ANOVA.

k) Abundance ranking of PB2 and SEMA4 family members based on average spectral counts among 398 identified adhesion/surface proteins across 122 treatment-naïve primary breast patient samples (https://doi.org/10.1016/j.cell.2020.10.036)[32]; the canonical binding ligands of PB2, SEMA4C, SEMA4D, and SEMA4A, are shown by yellow dots.

l) KM plot of distant metastasis-free survival (DMFS) of breast cancer patients based on mRNA expression of *SEMA4C*, p=0.0158, HR=1.69 (1.09-2.63) at the best cut off with N=122 for low expression and N=94 for high expression groups. The P-value was calculated by log-rank test.

m) KM plot of overall survival (OS) of breast cancer patients based on mRNA expression of *SEMA4C*, p=0.0104, HR=1.79 (1.02-3.15) at the best cut-off with N=986 for low expression and N=109 for high expression groups using TCGA BRCA data. The P-value was calculated by log-rank test.

Data are presented as mean values +/- SD in many panels, p-values reported are from two-sided unpaired t-tests unless specified. Source data are provided as a Source Data file.

Supplementary Figure S7

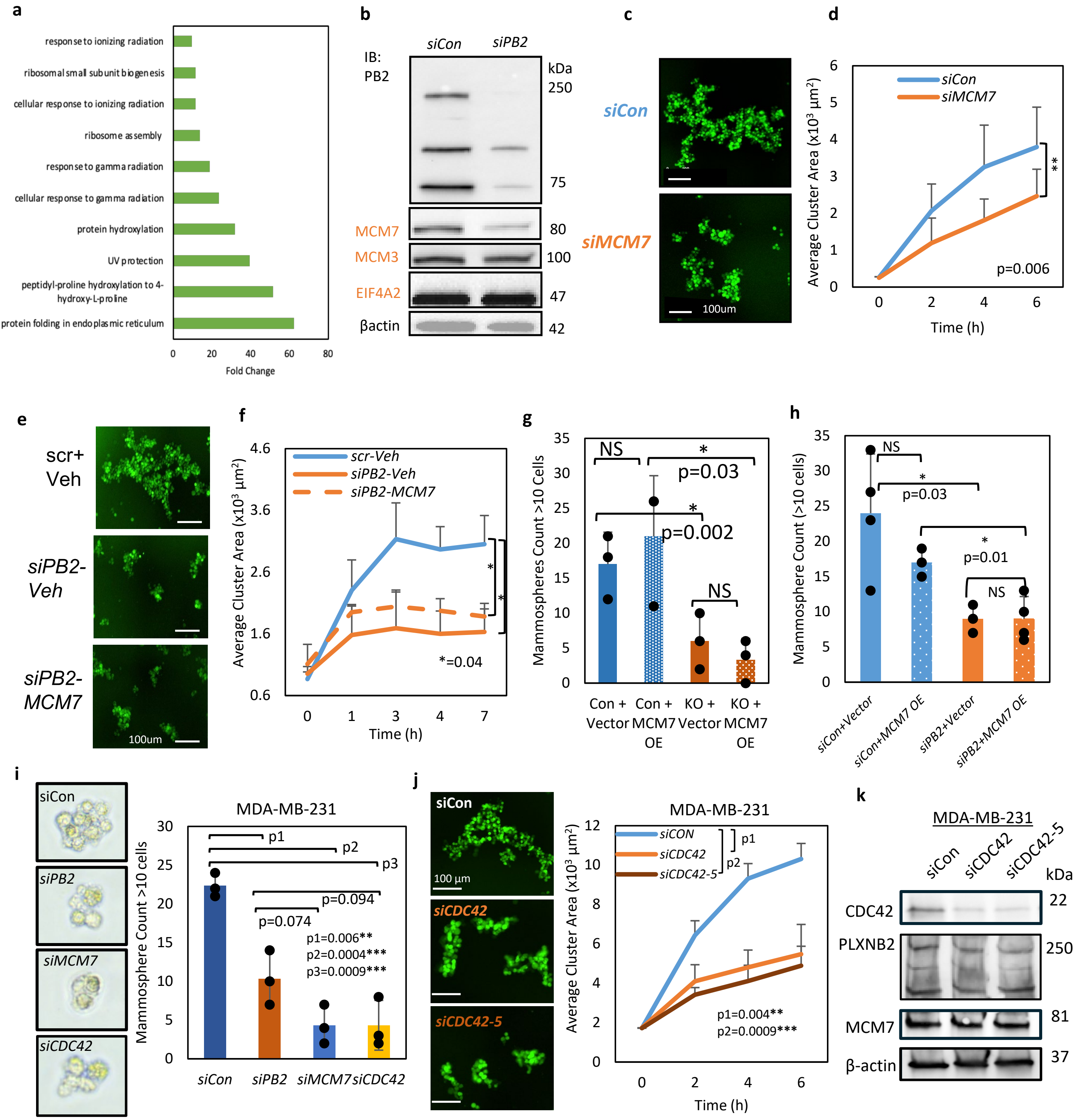

Supplementary Figure S7. PLXNB2-regulated pathways and potential downstream target MCM7.

a) GO biological processes analysis of altered proteins in Groups C and F in Fig. 3H, with clustering-specific up-regulation and down-regulation, respectively, and reversed in a *siPB2*-dependent manner.

b) Immunoblotting validation of top hits from Group B (see Fig. 3H) of global mass spectrometry analysis of si*PB2* single cells vs. clusters.

c-d) Representative images (d) and average cluster area (e) of MDA-MB-231 control vs. *MCM7* KD cells as taken by the IncuCyte. N=3 experiments with at least 3 technical replicates each.

e-f) Representative images (f) and average cluster area (g) MDA-MB-231 *PB2* KD cells with *MCM7* OE, N=3, images taken by Incuyte. *p=0.04.

g) Mammosphere formation of MDA-MB-231 *PB2* KO cells with *MCM7* OE compared to control after 8 days; mammospheres counted as groups of 10 or more cells, N=3. Control tumors vector vs *MCM7*, p= 0.57 (NS). *PB2* KO tumor cells vector vs *MCM7*, p = 0.27 (NS).

h) Mammosphere formation of MDA-MB-231 *siPB2* KD cells with *MCM7* OE compared to control after 8 days; mammospheres counted as groups of 10 or more cells, N=4. Control tumor cells vector vs MCM7, p = 0.22 (NS). *PB2* KD cells vector vs MCM7, p = 0.99 (NS).

i) Mammosphere images (left panel) and bar graph (right panel) of MDA-MB-231 cells transfected with siRNA control (*siCon*), *siPB2*, *siMCM7* and *siCDC42* showing *CDC42* knockdown (KD) similarly compromises mammosphere formation as *PB2* KD and *MCM7* KD.

j) Clustering images (left panel) and curves (right panel) of MDA-MB-231 cells transfected with siRNA control (*siCon*) and *siCDC42* showing *CDC42* depletion reduces tumor cell clustering efficiency.

k) Immunoblotting detection of reduced CDC42 in MDA-MB-231 cells after two consecutive transfections with *siCDC42* (smart siRNA pool) and *siCDC42-5* (individual siRNA) for gene KD.

Data are presented as mean values ± SD. P-values between two groups were calculated using two-sided unpaired t-tests. For comparisons involving more than two groups, one-sided ANOVA was used. Source data are provided as a Source Data file.

Supplementary Figure S8

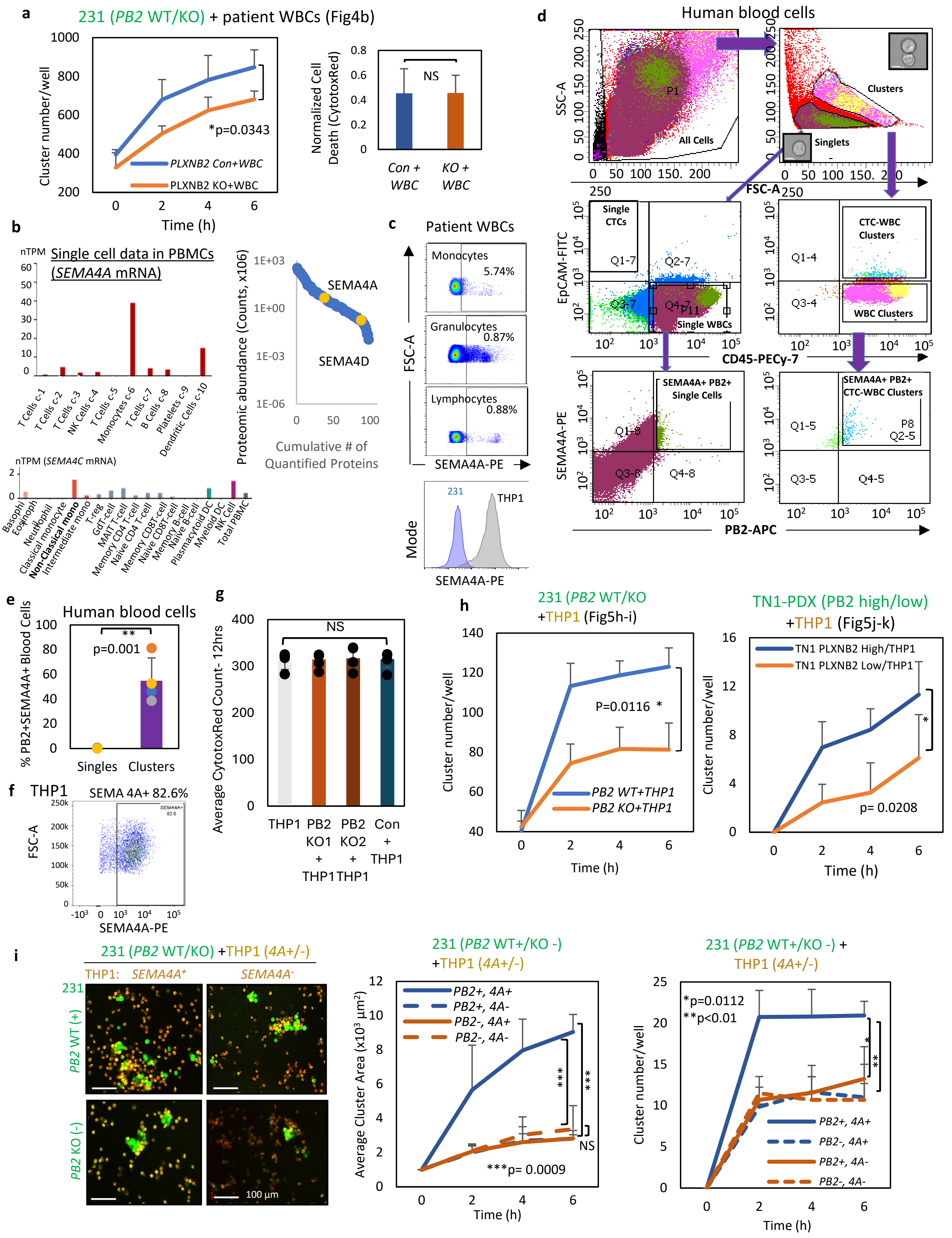

Supplementary Figure S8. SEMA4A expression in human WBCs and monocytes

a) Quantification of tumor cell-PBMC cluster numbers over time and normalized dead cell events (cytotoxic red dye-labeled) as measured by IncuCyte at 12 h during the MDA-MB-231 breast cancer cell co-culture with WBCs isolated from breast cancer patients, N=4. Data reported as mean +/-SD, p-value calculated from a two-sided unpaired t-test.

b) SEMA4A and SEMA4C expressions in human PBMCs (left panels) analyzed via single-cell sequencing of the Human Protein Atlas. Right panel: Abundance ranking of 96 identified adhesion/surface proteins in human monocytes (N=3), based on mass spectrometry (MS) quantitative analysis (https://doi.org/10.1038/s41598-020-61356-w)[68].

c) Top panels: Representative flow plots of SEMA4A expression in monocytes, granulocytes, and lymphocytes derived from the blood of patients with breast cancer. Bottom panel: Flow histogram of the SEMA4A (PE) expression levels in MDA-MB-231 (231) tumor cells and THP1 monocytes.

d) Representative gating strategy of flow cytometry analysis of advanced stage breast cancer patient WBCs stained for EpCAM, CD45, PB2, and SEMA4A to identify $PB2^{+}SEMA4A^{+}$ heterotypic CTC clusters.

e) Quantification of double positive $PB2^{+}SEMA4A^{+}$ clusters ($CD45^{+/-}$) in advanced stage breast cancer PBMCs, N=4 patients.

f) Flow cytometry analysis of PB2 and SEMA4A expression in THP1 monocyte cells, N=3.

g) Average Cytotox Red cell counts in heterotypic THP1 co-culture system as measured by IncuCyte, N=3.

h) Quantified numbers of heterotypic clusters of MDA-MB-231 or TN PDX tumor cells with THP1 monocytes (1:4) over 6 h, measured by Incucyte imaging, with N=3-5 experiments.

i) Representative images at 6 h (left panel) and cluster curves (size and numbers) of MDA-MB-231 WT (Con) or *PLXNB2* KO cells clustering with THP1 monocytes (sorted based on SEMA4C+/-) (4A+/- expression), N=3 experiments with at least 5 technical replicates each.

Data are presented as mean values ± SD. P-values between two groups were calculated using two-sided unpaired t-tests. For comparisons involving more than two groups, one-sided ANOVA was used. Source data are provided as a Source Data file.

Supplementary Figure S9

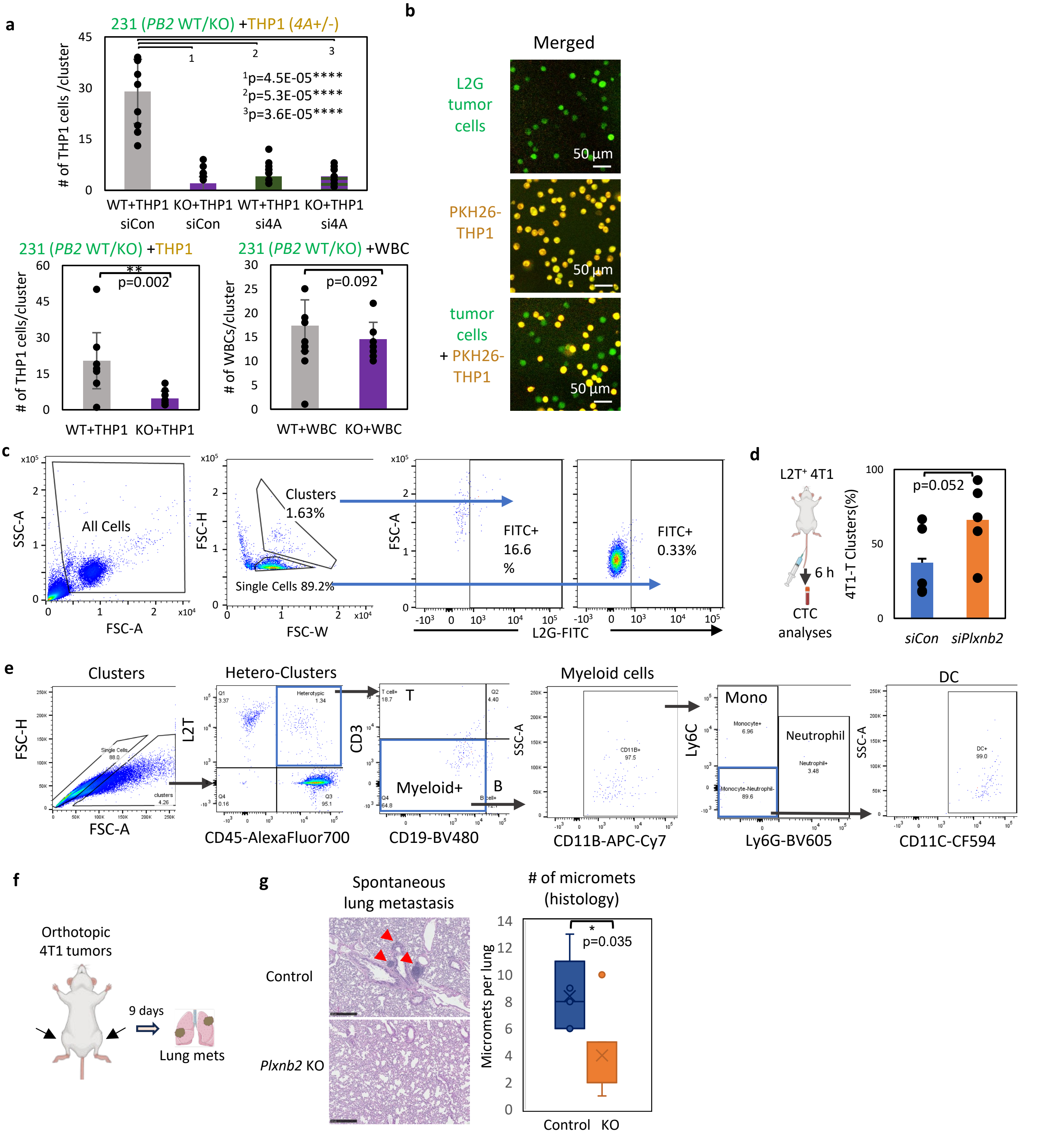

Supplementary Figure S9. Flow profiles of mouse blood cell clusters and quantified 4T1 CTC clusters after *Plxnb2* knockdown.

a) Number of immune cells (THP1 +/- si*SEMA4C* or WBCs) per cluster when mixed with MDA-MB-231 cells (*PB2* WT or KO), data derived from Fig 4L-M (top panel), Fig 4H-I (bottom left), and Fig 4B (bottom right panel), respectively.

b). Images of Luc2eGFP (L2G)-expressing tumor cells, PKH26-labeled THP1 cells, and mixed two cell types under merged channels (green and red) via IncuCyte microscopy imaging.

c) Representative flow analysis of L2G+ CTCs from mouse blood following 10 weeks of spontaneous metastasis of L2G+ MDA-MB-231 tumors, N=5.

d-e) Schematic and quantification (d) and flow gating (e) of PLXNB2 heterotypic mouse L2T+ 4T1 CTC clusters with T-cells, monocytes, or other WBCs at 6 h after tail vein injection of 4T1 mouse tumor cells transfected with siCon and si*Plxnb2*. 4T1 cells were transfected with siRNA for four knockdown cycles using RNAiMAX reagent to achieve >80% knockdown efficiencies. Then, $5x10^5$ 4T1 tumor cells were injected into Balb-c mice via the tail vein. Mice were sacrificed after 6 h for cardiac blood collection. RBCs were lysed, cells were stained for PLXNB2 and immune cell panels for flow cytometry, N=5 mice.

f) Schematic of 4T1 orthotopic tumor implants for analysis of spontaneous lung metastasis.

g) Representative H & E staining images of mouse lungs bearing spontaneous metastases of the Control and *Plxnb2* KO 4T1 tumors (left panels) and the counts of micrometastatic lesions in the mouse lungs of respective groups. Red arrows point to representative micrometastatic regions. Scale bar = 250 µm, and the p-value is calculated using Student's t-test with one-tailed distribution and two-sample unequal variance). Source data are provided as a Source Data file.
