# Supplementary material for: Computational ranking identifies Plexin-B2 in circulating tumor cell clustering with monocytes in breast cancer metastasis
Source: Nat Commun. 2025 Aug 16;16:7649. doi: 10.1038/s41467-025-62862-z (PMC12357858; doi:10.1038/s41467-025-62862-z)
Supplement: Supplementary file 2 — Description of Additional Supplementary Files [file 41467_2025_62862_MOESM2_ESM.pdf]

Description of Additional Supplementary Files

Supplementary Data 1: Mass Spec Ranking

Supplementary Data 2: TMA Patient Data

Supplementary Data 3: Global Mass Spec of Clusters

Supplementary Movie 1: Clustering assay shown in Fig. 3b (High)

Supplementary Movie 2: Clustering assay shown in Fig. 3b (Low)

Supplementary Movie 3: Clustering assay shown in Fig. 3d (siCon)

Supplementary Movie 4: Clustering assay shown in Fig. 3d (siPB2)

Supplementary Movie 5: Clustering assay shown in Fig. 3d (siPB2-10)

Supplementary Movie 6: Clustering assay shown in Fig. 3f (siCon)

Supplementary Movie 7: Clustering assay shown in Fig. 3f (siPB2)

Supplementary Movie 8: Clustering assay shown in Fig. 3f (siPB2-10)

Supplementary Movie 9: Clustering assay shown in Fig. 3j (Control)

Supplementary Movie 10: Clustering assay shown in Fig. 3j (PB2 knockout)

Supplementary Movie 11: Clustering assay shown in Fig. 3j (PB2 knockout + PB2 rescue)

Supplementary Movie 12: Clustering assay shown in Fig. 3j (PB2 knockout + mRDB mutant)

Supplementary Movie 13: Clustering assay shown in Fig. 3j (PB2 knockout + dVTDL mutant)

Supplementary Movie 14: Clustering assay shown in Fig. 3j (PB2 knockout + dECTO mutant)

Supplementary Movie 15: Clustering assay shown in Fig. 4f (Control + siCon)

Supplementary Movie 16: Clustering assay shown in Fig. 4f (Control + si4C)

Supplementary Movie 17: Clustering assay shown in Fig. 4f (KO + siCon)

Supplementary Movie 18: Clustering assay shown in Fig. 4f (KO + si4C)

Supplementary Movie 19: Clustering assay shown in Fig. 5h (PB2 WT)

Supplementary Movie 20: Clustering assay shown in Fig. 5h (PB2 KO)

Supplementary Movie 21: Clustering assay shown in Fig. 5j (PB2 high expression)

Supplementary Movie 22: Clustering assay shown in Fig. 5j (PB2 low expression)

Supplementary Movie 23: Clustering assay shown in Fig. 5l (PB2 WT + THP1 siCon)

Supplementary Movie 24: Clustering assay shown in Fig. 5l (PB2 WT + THP1 si4A)

Supplementary Movie 25: Clustering assay shown in Fig. 5l (PB2 KO + THP1 siCon)

Supplementary Movie 26: Clustering assay shown in Fig. 5l (PB2 KO + THP1 si4A)

Supplementary Movie 27: Clustering assay shown in Fig. S5a (siCon)

Supplementary Movie 28: Clustering assay shown in Fig. S5a (siPB2-11)

Supplementary Movie 29: Clustering assay shown in Fig. S5h (siCon)

Supplementary Movie 30: Clustering assay shown in Fig. S5h (siPB2)

Supplementary Movie 31: Clustering assay shown in Fig. S5k (siCon)

Supplementary Movie 32: Clustering assay shown in Fig. S5k (siPB2)

Supplementary Movie 33: Clustering assay shown in Fig. S5k (siPB2-10)

Supplementary Movie 34: Clustering assay shown in Fig. S6a (Control)

Supplementary Movie 35: Clustering assay shown in Fig. S6a (KO1)

Supplementary Movie 36: Clustering assay shown in Fig. S6a (KO2)
