# Supplementary figures and images for: Computational ranking identifies Plexin-B2 in circulating tumor cell clustering with monocytes in breast cancer metastasis
Source: Nat Commun. 2025 Aug 16;16:7649. doi: 10.1038/s41467-025-62862-z (PMC12357858; doi:10.1038/s41467-025-62862-z)

**Fig 3i**

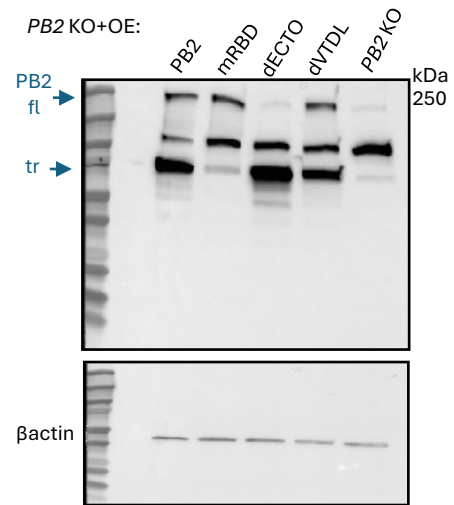

**Fig 4b**

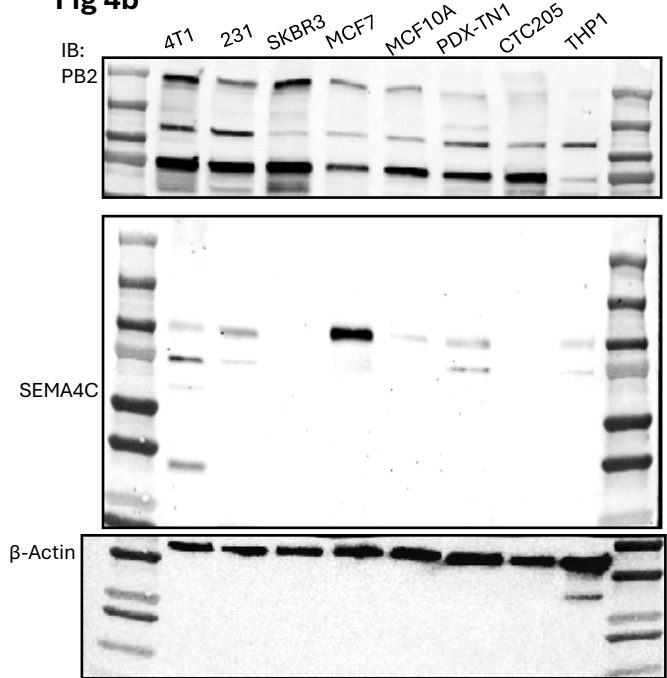

**Fig 4c (IP)**

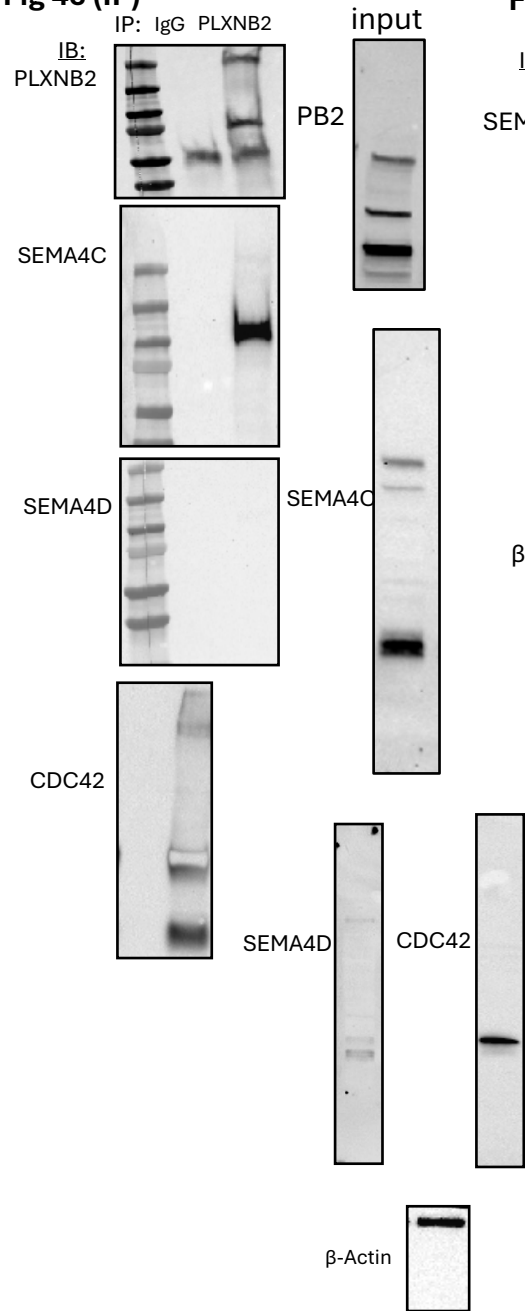

**Fig 4d**

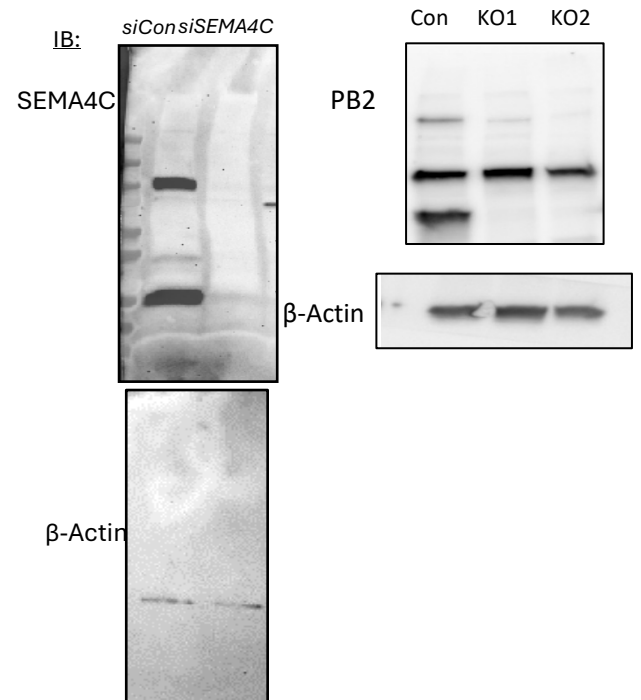

**Fig 4e**

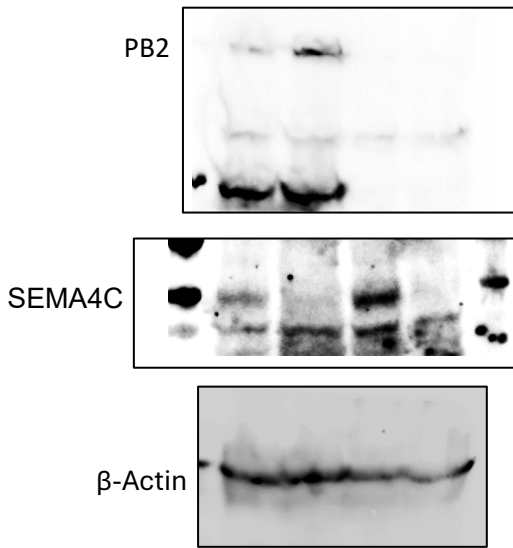

**Fig 5f (IP)**

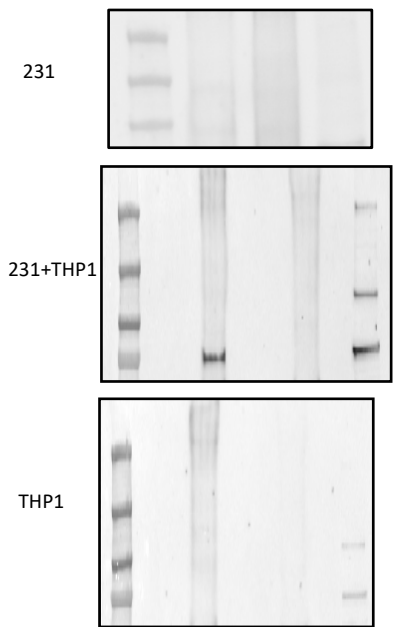

**Fig 5g (IP)**

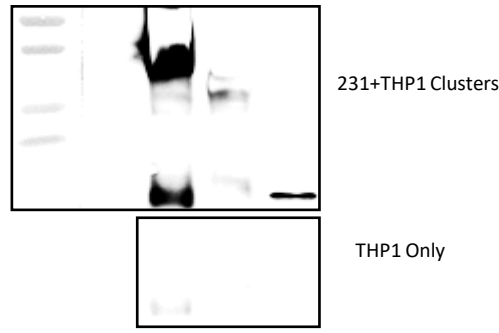

**Fig S1e**

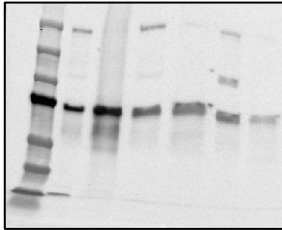

**Fig S2d**

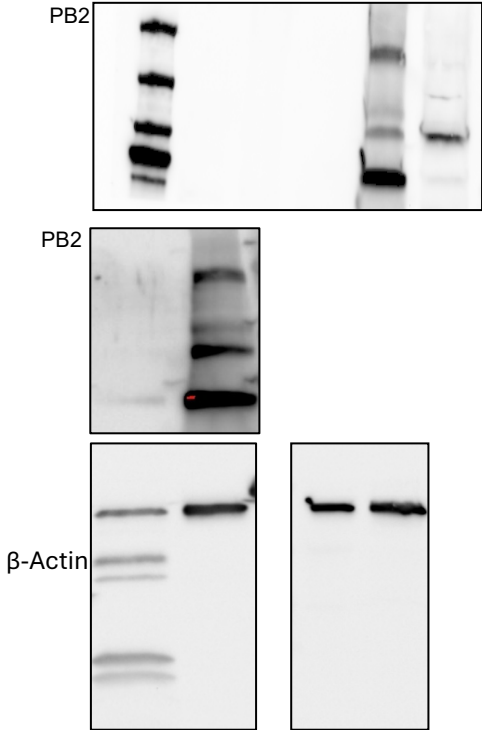

**Fig S4a**

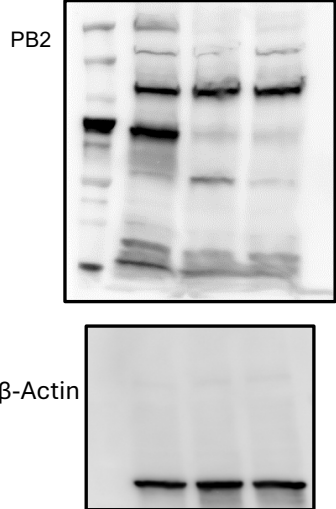

**Fig S4g**

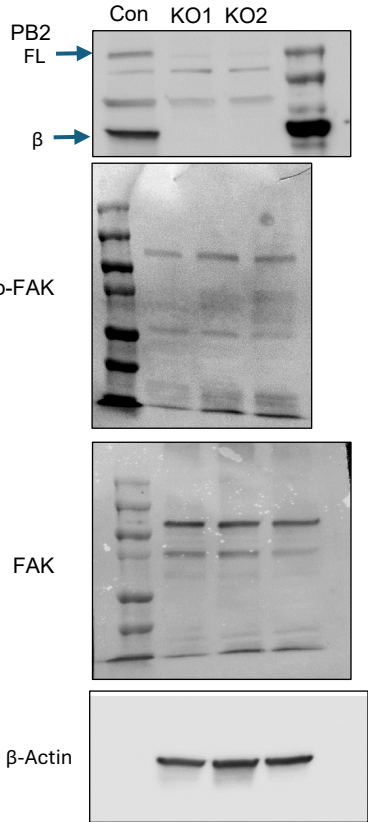

**Fig S5a**

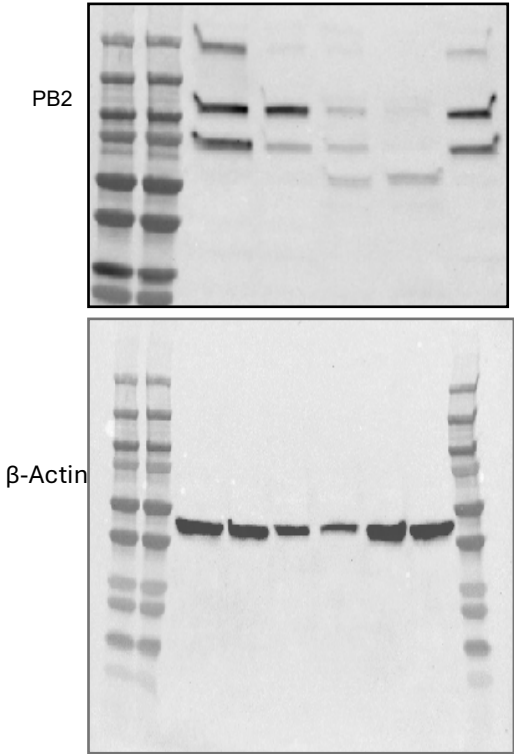

**Fig S7b**

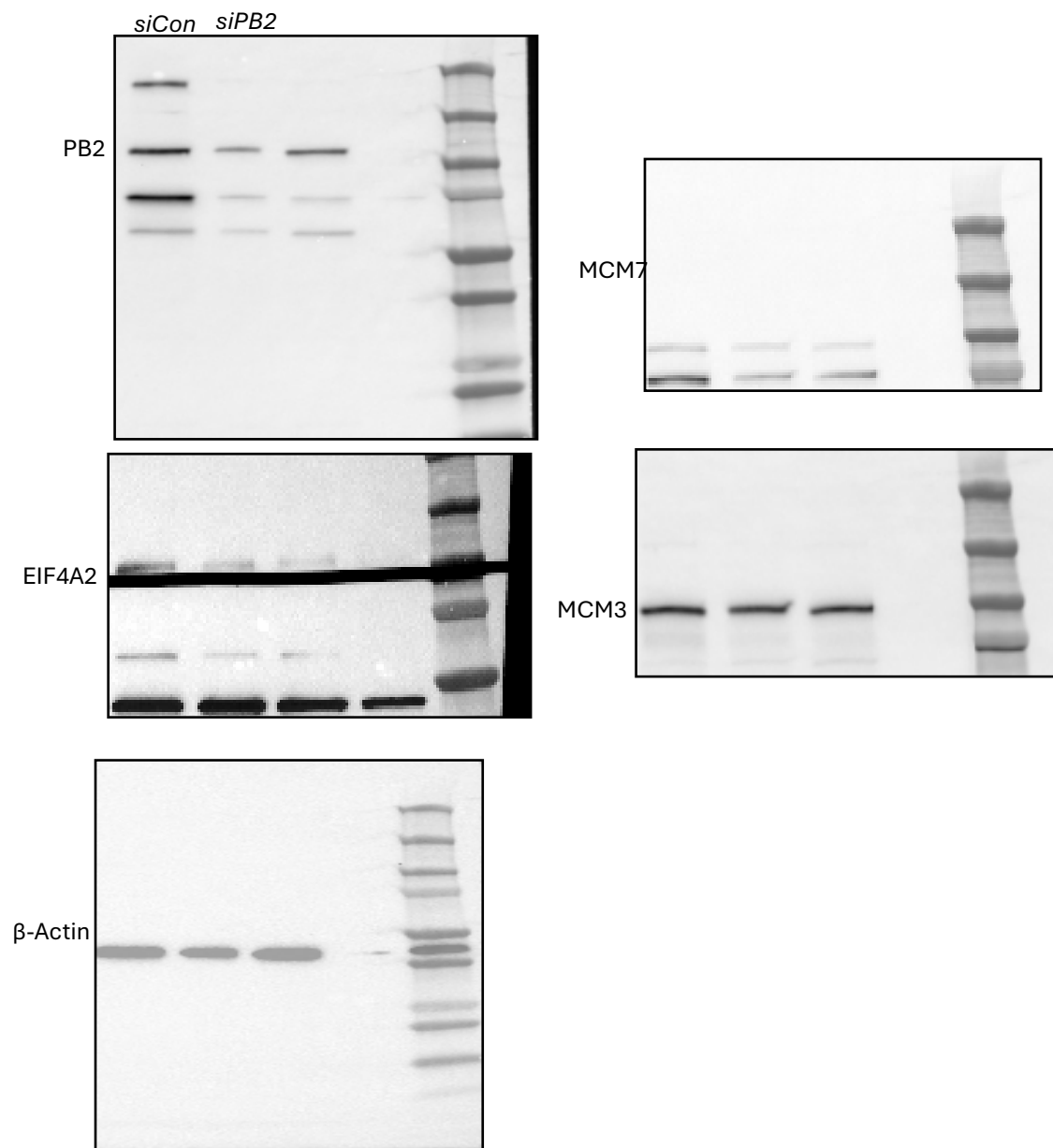

**Fig S7k**

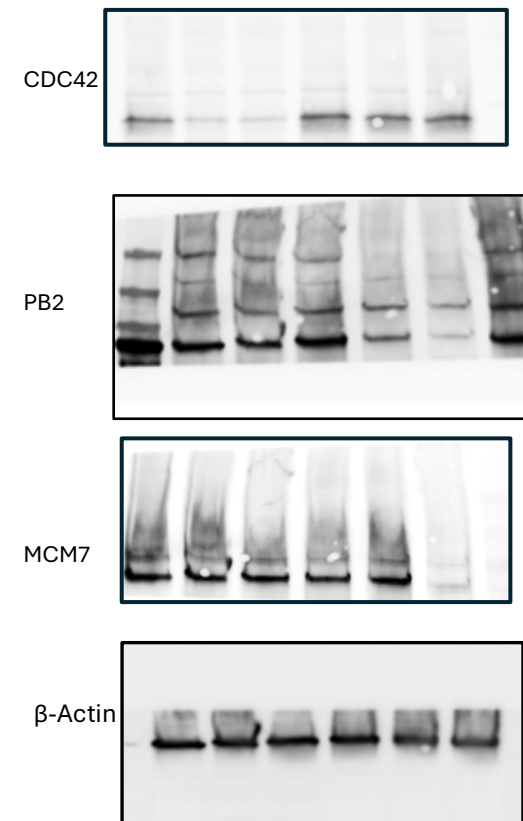

Supplement: Supplementary file 44 — Source Data [file 41467_2025_62862_MOESM44_ESM.zip › Uncropped source blots.pdf]
